# A 3D-printed anatomical pancreas and kidney phantom for optimizing SPECT/CT reconstruction settings in beta cell imaging using ^111^In-exendin

**DOI:** 10.1186/s40658-016-0165-0

**Published:** 2016-12-07

**Authors:** Wietske Woliner-van der Weg, Laura N. Deden, Antoi P. W. Meeuwis, Maaike Koenrades, Laura H. C. Peeters, Henny Kuipers, Geert Jan Laanstra, Martin Gotthardt, Cornelis H. Slump, Eric P. Visser

**Affiliations:** 1 Department of Radiology and Nuclear Medicine, Radboud University Medical Center, P.O. Box: 9101, 6500 HB Nijmegen, The Netherlands; 2 Department of surgery, Rijnstate Hospital, Arnhem, The Netherlands; 3 Department of Robotics and Mechatronics, MIRA Institute for Biomedical Technology and Technical Medicine, University of Twente, Enschede, The Netherlands; 4 Department of Vascular Surgery, Medical Spectrum Twente, Enschede, The Netherlands; 5 Department of Rehabilitation, Radboud University Medical Center, Nijmegen, The Netherlands

**Keywords:** 3D printing, Beta cell imaging, Quantification, SPECT, ^111^In-exendin

## Abstract

**Background:**

Quantitative single photon emission computed tomography (SPECT) is challenging, especially for pancreatic beta cell imaging with ^111^In-exendin due to high uptake in the kidneys versus much lower uptake in the nearby pancreas. Therefore, we designed a three-dimensionally (3D) printed phantom representing the pancreas and kidneys to mimic the human situation in beta cell imaging. The phantom was used to assess the effect of different reconstruction settings on the quantification of the pancreas uptake for two different, commercially available software packages.

**Methods:**

3D-printed, hollow pancreas and kidney compartments were inserted into the National Electrical Manufacturers Association (NEMA) NU2 image quality phantom casing. These organs and the background compartment were filled with activities simulating relatively high and low pancreatic ^111^In-exendin uptake for, respectively, healthy humans and type 1 diabetes patients. Images were reconstructed using Siemens Flash 3D and Hermes Hybrid Recon, with varying numbers of iterations and subsets and corrections. Images were visually assessed on homogeneity and artefacts, and quantitatively by the pancreas-to-kidney activity concentration ratio.

**Results:**

Phantom images were similar to clinical images and showed comparable artefacts. All corrections were required to clearly visualize the pancreas. Increased numbers of subsets and iterations improved the quantitative performance but decreased homogeneity both in the pancreas and the background. Based on the phantom analyses, the Hybrid Recon reconstruction with 6 iterations and 16 subsets was found to be most suitable for clinical use.

**Conclusions:**

This work strongly contributed to quantification of pancreatic ^111^In-exendin uptake. It showed how clinical images of ^111^In-exendin can be interpreted and enabled selection of the most appropriate protocol for clinical use.

## Background

The development of a non-invasive, imaging-based method for quantification of the beta cell mass could greatly enhance our understanding of the complex pathophysiology underlying the development of diabetes. Therefore, we have studied the use of pancreatic uptake of ^111^In-exendin as an imaging biomarker for the beta cell mass preclinically, and recently also in type 1 diabetes (T1D) patients and healthy humans [1]. Pancreatic uptake of ^111^In-exendin is low compared to the uptake in the nearby kidneys and strongly varies amongst healthy humans, in which at least a factor seven of difference in pancreatic uptake was observed [2]. This is in line with the expected variation in beta cell mass. The average uptake in T1D patients is even two and a half times lower than in healthy volunteers [2]. In order to use this tracer as a biomarker for beta cell mass, reliable quantification of the uptake is required.

To improve quantification of pancreatic ^111^In-exendin uptake in this clinical study, a phantom mimicking the human situation was required. Quantitative single photon emission computed tomography (SPECT) is challenging in general [3–5], mostly due to the relatively low detection efficiency as compared to positron emission tomography (PET), and the more complicated attenuation correction. With the use of ordered subset expectation maximization (OSEM) reconstruction and adequate correction methods, quantification of SPECT images is currently viable and attractive [4], and it enables quantification of gamma-emitting radionuclides, like ^111^In. Imaging and quantification of the pancreatic beta cells with ^111^In-exendin is even more challenging, due to the high uptake in the nearby kidneys versus the low uptake in the pancreas [1].

The initial reconstructions in this clinical study [1] were made with the SPECT/CT software that we generally use in the clinic, Syngo Flash 3D (Siemens Medical, Erlangen, Germany). This reconstruction algorithm led to a low count rim surrounding the kidneys that should most likely be considered as an artefact. Thereafter, we used another available reconstruction package: Hybrid Recon oncology (Hermes Medical Solutions, Stockholm, Sweden). This reconstruction algorithm also led to image features that should most probably be considered as artefacts; a low count rim surrounding the kidneys was also present, similar to Flash 3D, but less pronounced, and a hotspot in the spinal area was observed. These features are shown in Table 1.

**Table 1:** Initial findings with reconstruction settings of clinical scans

	Flash 3D	Hybrid Recon
Version	Siemens Syngo MI.SPECT application E.soft 2009a, 8.1.15.7 service pack 2	HERMES P5 GOLD 4.6A Hybrid Recon 1.1.2
Reconstruction algorithm	OSEM3D	OSEM3D, MAP_MRP_ and MAP_smoothing_
# iterations–# subsets	6–16	4–16
Attenuation correction	CT-based	CT-based
Scatter correction	Triple energy window, SPECT-based	CT, Monte Carlo-based
Both photopeaks of ^111^In reconstructed	Yes	Yes
Collimator correction	Mathematically calculated 3D cone beam modelling	Mathematically calculated 3D cone beam modelling
Post reconstruction filtering	Gaussian FWHM = 0.84 cm	Gaussian FWHM = 0.96 cm
Most obvious artefacts in the initial human images	Large low-intensity rim around kidneys	Small low-intensity rim around the kidneys, somewhat increased intensity in the vertebrae area

As already mentioned, adequate correction methods are a key for quantitative use of SPECT images. Commercial SPECT (CT) reconstruction algorithms have been developed for general purpose imaging, and not specifically to quantify low uptake in a specific organ (pancreas), with high activity in nearby organs (kidneys). The origin of artefacts, their influence on quantification, and sometimes even the presence of artefacts cannot reliably be investigated in human, because the true distribution of the radiotracer is unknown. Also, details about the working of commercially available reconstruction software are not always available to the user; partially, the software is a black box. Therefore, the use of phantoms is of major importance for recognizing and understanding the origin of reconstruction artefacts, and in our case, also to enable reliable quantification in beta cell imaging. Normally, phantoms in nuclear medicine imaging consist of simple geometrical shapes such as spheres, cylinders and rods. However, these are less suitable to mimic the ^111^In-exendin distribution in the pancreas and kidneys in humans. Therefore, in this study, an anatomical phantom was developed and used.

In the last few years, 3D printing has become an established method for fast and affordable production of advanced and customized phantoms for medical imaging [6–10]. We employed this technique to create an anatomical phantom containing a pancreas, two kidneys and a background region for optimizing the SPECT/CT reconstructions to allow quantification of the pancreatic uptake of ^111^In-exendin in the clinical study [1].

The objective of this work was to mimic human ^111^In-exendin imaging with this custom-made anatomical phantom to clarify how the human images should be interpreted (e.g. whether artefacts play a role). This is important in visualization and quantification of the low pancreatic uptake, since this is challenged by relatively high kidney activity. Using the phantom, we assessed different reconstruction settings for two different commercially available software packages and selected the most suitable reconstruction protocol for visualization and quantification of pancreatic ^111^In-Exendin uptake.

## Methods

We designed and printed 3D anatomical inserts representing the pancreas and kidneys that can be placed within the casing of the National Electrical Manufacturers Association (NEMA) NU2 image quality body phantom [11] after removing the standard spheres and cylinder inserts.

This anatomical phantom was filled with known activity concentrations to simulate SPECT imaging of T1D patients (low pancreatic uptake) and healthy humans (higher pancreatic uptake).

All SPECT/CT images were acquired using an integrated SPECT/CT scanner (Symbia T16, Siemens Healthcare, Molecular Imaging, Hoffman Estates, IL, USA) equipped with parallel-hole medium-energy collimators. Software of the before mentioned two vendors (Flash 3D, Hybrid Recon) and different reconstruction settings were tested to find the optimal algorithm and settings. After evaluation of the phantom results, the optimal reconstruction protocol was applied to human data of the clinical study. Details about the phantom development, the experiments and image analysis are described below.

### Phantom development

The pancreas and kidney organ models and their relative positions were based on an MRI dataset of a male patient of 87 kg. Forty sequential T2 weighted HASTE images were acquired with a Siemens Magnetom TrioTim 3.0 Tesla MRI scanner as part of another clinical study. The volumes and distances between the organs measured in the MRI images were compared to those in CT images of humans imaged with ^111^In-exendin, to make sure the anatomy was representative for our study population.

Images were processed in Mimics v.14.0 (Materialise HQ, Leuven, Belgium). Solid 3D models of the kidneys and the pancreas were created from segmentations that were performed on the axial images. The models were smoothed with a first-order Laplacian (70 iterations, smooth factor 0.9) and exported as 3D triangular surface meshes (STL-files). Using the open source software Meshlab (v.1.3.2, http://meshlab.sourceforge.net/), the surfaces were further processed to create a realistic anatomical model; abnormal bulges and sharp edges were removed or smoothed and holes were filled. The solid bodies were transformed to hollow organ models with a wall thickness of 4 mm with Meshlab and SolidWorks (v. 2012, Dassault Systèmes SolidWorks Corp., Waltham, MA, USA), and flanges were added for fixation of the organs. Then, the organ models were 3D-printed with an Objet Eden250™ printer (Stratasys, Eden Prairie, MN, USA) based on ultra-thin layer PolyJet™ technology. Printer specifications include a layer thickness of 16 μm, tray size of 260 × 260 × 200 mm and accuracy of 100 μm. The material used for printing was the transparent standard plastic VeroClear RGD810, which is comparable with PMMA, which has a polymerized density of 1.18–1.19 g/cm^3^ and Z_eff_ = 6.56 [12]. This is in the same range as soft tissue with an Z_eff_ = 6.35 [12]. The non-soluble support material was taken out by hand.

**Fig. 1:**
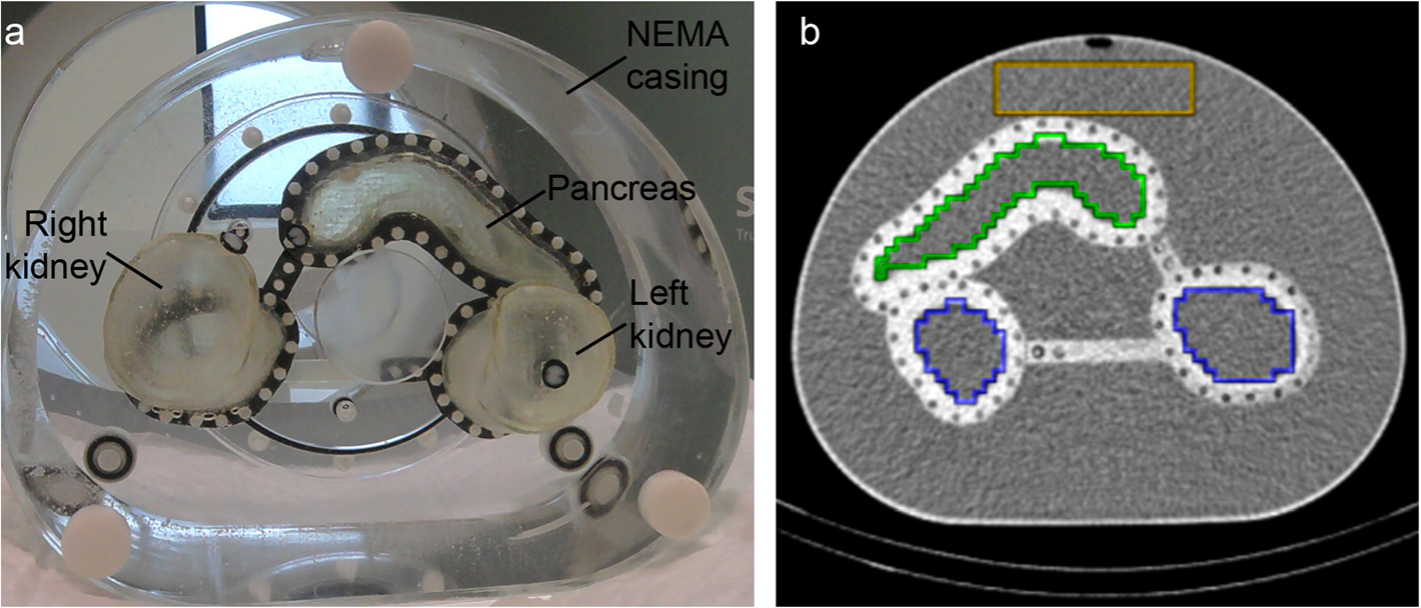
**a** A photograph of the 3D-printed pancreas and kidney compartments in the NEMA-NU2 image quality phantom casing. **b** A CT cross-section of the phantom showing the regions of interest in the kidneys, pancreas and background

To enable filling with radioactive solution, holes were drilled in the organs, in which nylon screws sealed with rubber O-rings were placed. The printed organs were placed within the NEMA casing and fixated with screw threads in the original screw holes in the lid of the NEMA casing (Fig. 1).

The volumes of the pancreas and kidney inserts were measured by weighing the organs empty and filled with water. The volume of the background in the NEMA casing was measured by filling it with water with a graduated cylinder.

### Reconstruction software

Reconstruction software Flash 3D and Hybrid Recon were used. In both software packages, a 3D ordered subset expectation maximization (OSEM3D) algorithm was available. In Hybrid Recon also, a maximum a posteriori (MAP) algorithm with a smoothing prior (MAP_smoothing_) and with a median root prior (MAP_MRP_) was available. With the smoothing prior, it is hypothesized that the pancreas activity is more homogeneously distributed in the images. The MAP_MRP_ preserves edges and hotspots more than the smoothing prior but also assumes a locally monotonic image [13]. Scatter, CT map-based attenuation and collimator correction were applied as available in the reconstruction software. Reconstruction settings are given in the experiment descriptions (‘Phantom experiment 1’ and ‘Phantom experiment 2’), and more details about the reconstruction software and methods are given in Table 1.

### Phantom experiment 1—high pancreas activity (simulating healthy individuals)

This first experiment aimed to investigate the different reconstruction software and settings in a simulation of ^111^In-exendin imaging in non-diabetic, healthy human (relatively high pancreas uptake compared to T1D patients). Activity concentrations for the pancreas, kidneys and background in the phantom were based on an estimation of the uptake of ^111^In-exendin in Flash 3D reconstructions of the first three healthy humans from the clinical study [1]. As a measure for the uptake, the number of reconstructed counts in the pancreas, kidneys and background of these humans was retrieved by delineating the pancreas and kidneys and a background volume using the Inveon Research Workplace (IRW) software (Siemens Inc, Munich, Germany). The corresponding amount of ^111^In activity to be inserted into the phantom compartments was calculated by multiplying these intensities with a calibration factor determined from SPECT imaging of a cylindrical phantom with the same acquisition and reconstruction settings as the human SPECT scans. The pancreas-to-kidney activity concentration ratio was 1:57, the pancreas-to-background ratio 1:0.005. Table 2 provides the volumes of the compartments and the amount of ^111^In activity within the compartments. To avoid sticking of ^111^In to the phantom wall, 0.05% Tween was added to the solution.

**Table 2:** Phantom and filling characteristics

		Pancreas	Left kidney	Right kidney	Background	Pancreas-to-kidney ratio
	Volume (ml)	82	206	222	9.26 × 10^3^	
Experiment 1	Activity concentration ^111^In at start time of the scan (MBq/ml)	3.94 × 10^−3^	0.226	0.226	2.11 × 10^−5^	1:57
	Total activity ^111^In at scan time (MBq)	0.32	46.6	50.2	0.20	
Experiment 2	Activity concentration ^111^In at scan time (MBq/ml)	1.93 × 10^−3^	0.209	0.209	2.10 × 10^−5^	1:108
	Total activity ^111^In at scan time (MBq)	0.16	43.1	46.5	0.19	

The acquisition protocol for the phantom imaging was the same as used for humans imaged with ^111^In-exendin at our department [1]. To avoid possible incorrect handling of different detector-to-object distances and disturbing quantitative use of the images, the phantom was scanned with a fixed radius of rotation (25 cm), a fixed bed height (13 cm) and 128 views in step-and-shoot mode (64 views per camera head) with an acquisition time of 40 s per view. Symmetric 15% energy windows over the 171 and the 245 keV photopeaks were used, with additional 7% lower and upper scatter windows, resulting in a total of six energy windows per scan.

To determine the optimal number of iterations and subsets in the reconstruction protocols, different settings were applied, in OSEM3D, based on the available combinations; 6/16 (6 iterations and 16 subsets), 12/16, 12/32, 18/16, 18/32, 24/32, 30/32 and 32/32 for Hybrid Recon were chosen and 3/16, 6/16, 6/62, 12/16, 12/32, 18/16, 18/32, 24/16, 24/32, 30/16 and 30/32 for Flash 3D. Preliminary tests showed that quantification improved with increasing the numbers of iterations and subsets. We therefore used higher numbers compared to what is commonly applied in clinical use (knowing this would increase inhomogeneity).

The analysis of the results was performed as will be described in the subsection ‘phantom image analysis’.

### Phantom experiment 2—low pancreas activity (simulating T1D patients)

The second experiment was performed for further verification of the findings of the first experiment, with experimental settings that simulate the T1D patients we imaged with ^111^In-exendin. In T1D patients, the pancreas uptake is even lower than in healthy human. Visualization of the lower uptake is even more demanding regarding the reconstruction protocol. Therefore, for further investigation of reconstruction details, we chose to use this T1D simulation instead of the simulation of a healthy situation in experiment 1.

Phantom filling was based on measurements in T1D patients of the clinical study [1]. The pancreas-to-kidney activity concentration ratio was 1:108, the pancreas-to-background ratio 1:0.01. Activity concentrations in each compartment are shown in Table 2. Acquisition was performed with the same settings as in experiment 1. In the first experiment, reconstructions performed with Hybrid Recon led to more representative activity ratios (see results, ‘Activity concentration ratios in the phantom images’). Therefore, Hybrid Recon was used for reconstruction of the data in this second experiment, and more reconstruction settings were examined. Not only OSEM3D but also MAP_smoothing_ and MAP_MRP_ reconstructions were made. The MAP_smoothing_, MAP_MRP_ and OSEM3D images were reconstructed with three sets of iterations and subsets: 6/16, 12/16 and 18/32.

After analysing these different reconstruction methods, we also paid attention to the effect of several correction settings (scatter correction (SC), attenuation correction (AC) and collimator correction (CC)) on the pancreas visualization and quantification. Reconstructions using OSEM3D were performed with all corrections, with CC and AC, with only CC, and without corrections. Other correction combinations were not available in the software.

### Phantom image analysis

All phantom images were visually assessed, and intensities of the different compartments were determined, to decide which reconstruction software and settings lead to the best images for quantification of the ^111^In-exendin uptake in the pancreas.

Visual assessment focussed on homogeneity within the pancreas and kidneys, on artefacts, and on the position of the pancreas in the CT images compared to the position in the SPECT images.

For quantitative analysis, pancreas and kidneys were manually delineated as volumes of interest (VOI) on CT using the IRW software. In the background, a box-shaped VOI was defined anterior of the pancreas compartment (Fig. 1b). As a measure for the uptake, the average number of reconstructed counts per voxel was retrieved for all VOIs. In order to keep it concise, in this manuscript we call the number of reconstructed counts per voxel, ‘intensity’.

Recovery of activity was calculated as the measured intensity (counts/ml) divided by the real activity concentration (Bq/ml). Also, pancreas-to-background and pancreas-to-kidney intensity ratios were calculated and compared to the real activity concentration ratios.

### Verification of phantom-based optimization on human images

As a final test, the optimized reconstruction protocol determined from the phantom experiments will be applied to data of the clinical study [1]. These images were acquired with the same acquisition settings as both phantom experiments and were acquired 24 hours after injection, within the same imaging session as the low-dose CT. SPECT imaging took about 45 min.

## Results

**Fig. 2:**
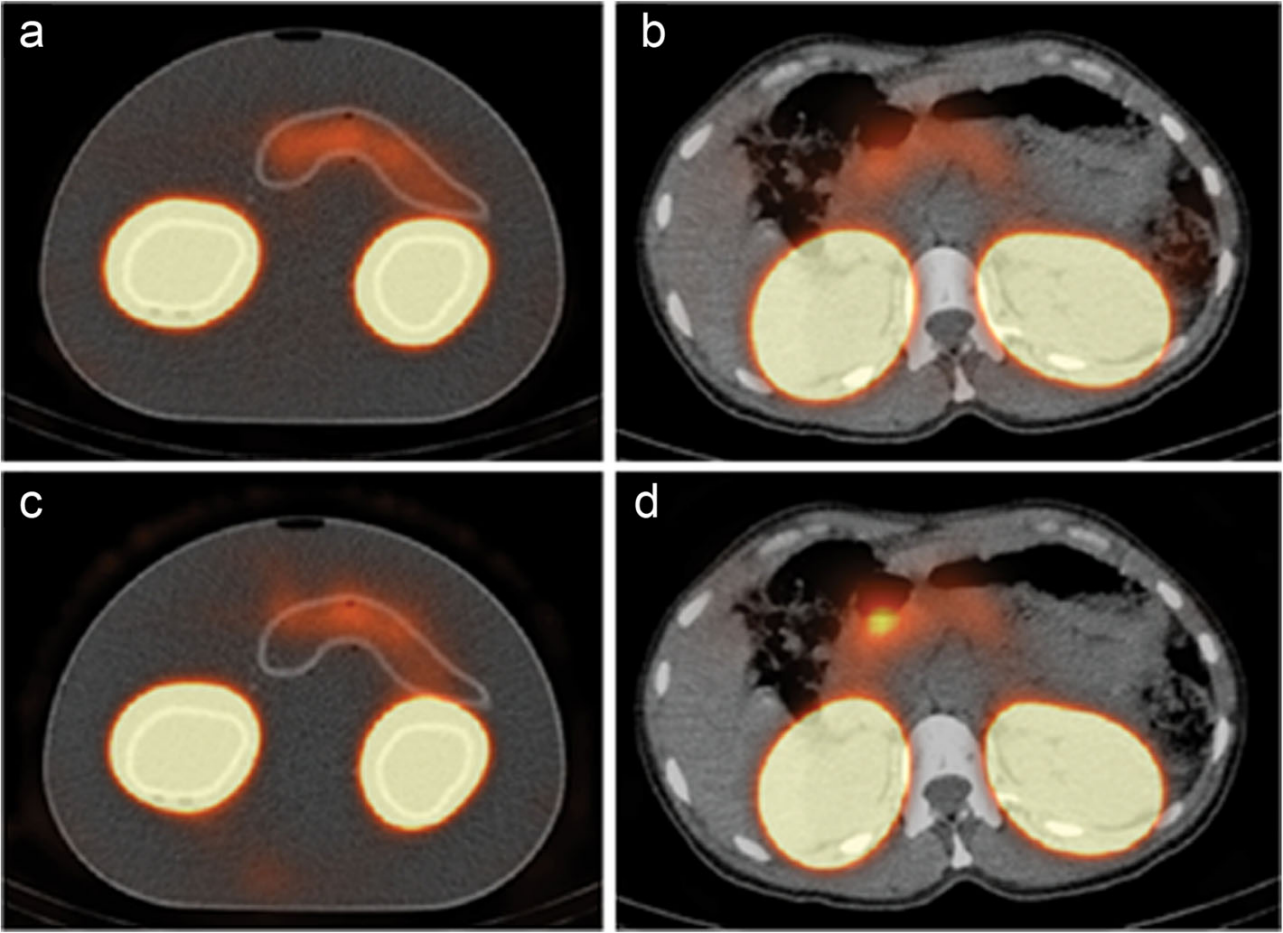
Reconstructed SPECT/CT images of the phantom from experiment 1 (**a**, **c**) and a healthy human (**b**, **d**). **a**, **b** Images made with Hybrid Recon OSEM3D and **c, d** Flash 3D OSEM3D images. All reconstructions were performed with 6 iterations and 16 subsets and used attenuation, scatter, and collimator correction

SPECT imaging of the 3D-printed phantom (Fig. 1) led to images that were similar to human images in terms of visualization of the pancreas and kidneys and artefacts (Fig. 2).

### Homogeneity and intensity within the 3D-printed organs

**Fig. 3:**
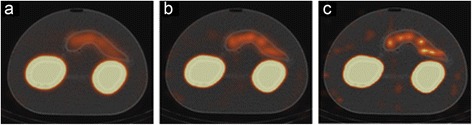
The effect of increasing the number of iterations: image **a** was made with 6 iterations and 16 subsets, **b** with 12 iterations and 32 subsets and **c** with 32 iterations and 32 subsets. All images were reconstructed with Hybrid Recon OSEM3D with attenuation, collimator and scatter correction. Window and level settings are the same in all images and were chosen so that the pancreas was visible

Increasing the number of iterations or subsets led to noisier images, which resulted in more spots in the pancreas (Fig. 3 for Hybrid Recon, Flash 3D gives similar results). In humans, these spots in the pancreas could easily be misinterpreted as inhomogeneous uptake of ^111^In-exendin, which could be related with the patient having an insulinoma.

In Flash 3D reconstructions, the intensity in the tail and the edge of the head of the pancreas (close to the kidney) was lower compared to that in the other parts of the pancreas. Also, it seems that the pancreas position on SPECT is more towards anterior compared to CT (Fig. 2c). In Hybrid Recon, the position of the pancreas is similar in SPECT and CT, and the intensity was more homogeneous across the entire pancreas. For a high number of iterations (18 iterations, 32 subsets) in Hybrid Recon, the intensity distribution in the pancreas was more homogeneous with the MAP reconstructions compared with the OSEM3D reconstruction.

**Fig. 4:**
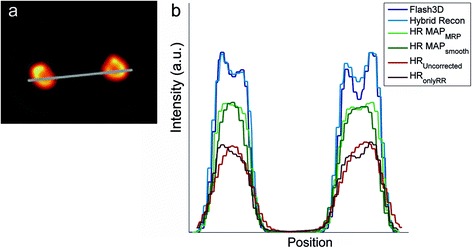
**a** The Hybrid Recon OSEM3D reconstruction of the phantom with attenuation, collimator and scatter correction, 6 iterations and 16 subsets. The window/level was scaled such that variations in intensity in the kidneys were visible. **b** Line profiles through the kidneys (line positioned as in **a** for a Hybrid Recon and Flash 3D reconstruction with 6 iterations and 16 subsets, attenuation scatter and collimator correction

In both Flash 3D and Hybrid Recon, the intensity in the kidneys is high and not homogeneously distributed (see ‘Artefacts in the phantom images’ and Fig. 4a). However, the intensity was similar for the different reconstruction settings within Flash 3D as well as within Hybrid Recon.

**Fig. 5:**
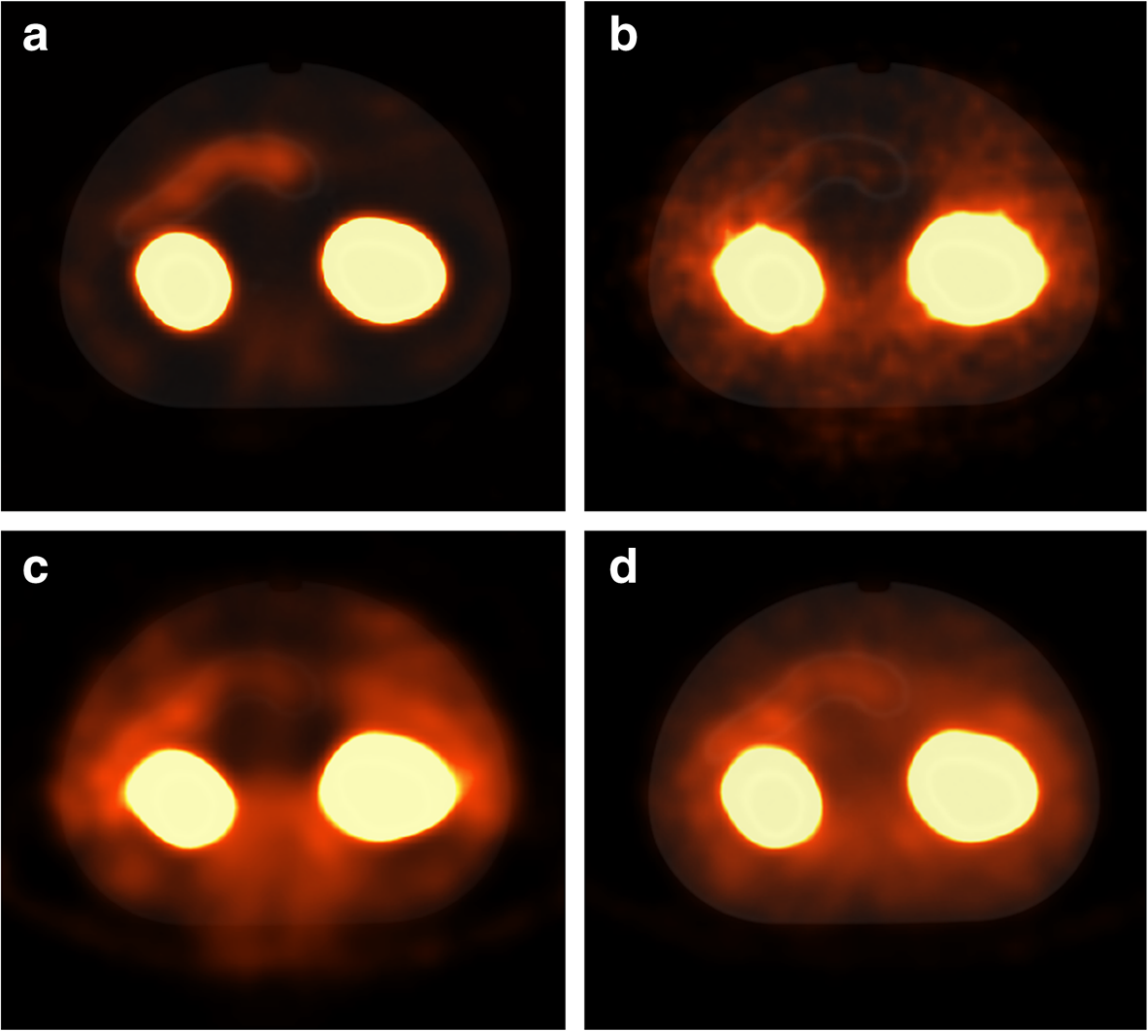
Images from the second experiment, all reconstructed using HybridRecon OSEM3D with 6 iterations and 16 subsets and different corrections. In **a**, collimator, attenuation and scatter correction was applied; in **b**, no corrections were applied; in **c**, only collimator correction was applied; and in **d**, collimator and attenuation correction was applied. Activity in the pancreas was only observed well if all corrections were applied

The effect of the corrections was tested with Hybrid Recon OSEM3D and MAP_MRP_ with 6 iterations and 16 subsets in the second experiment. As default, we used all the corrections. Only to investigate the effect of corrections on the images, we limited the use of corrections and made several reconstructions. As expected, AC results in a large increase in the number of counts in the whole phantom. Notably, applying the SC on top of the AC and CC influences the low intensity areas (pancreas and background) more than the high intensity areas (kidneys). In the kidneys, the measured activity concentration decreased by 15%, while a decrease of 71% and 88% was seen in the pancreas and the background, respectively. The reconstructed images with the available correction combinations (CC + AC + SC, none, CC and CC + AC) are shown in Fig. 5. All three corrections were required to be able to identify the pancreas in the second experiment, simulating the low pancreas activity.

### Artefacts in the phantom images

**Fig. 6:**
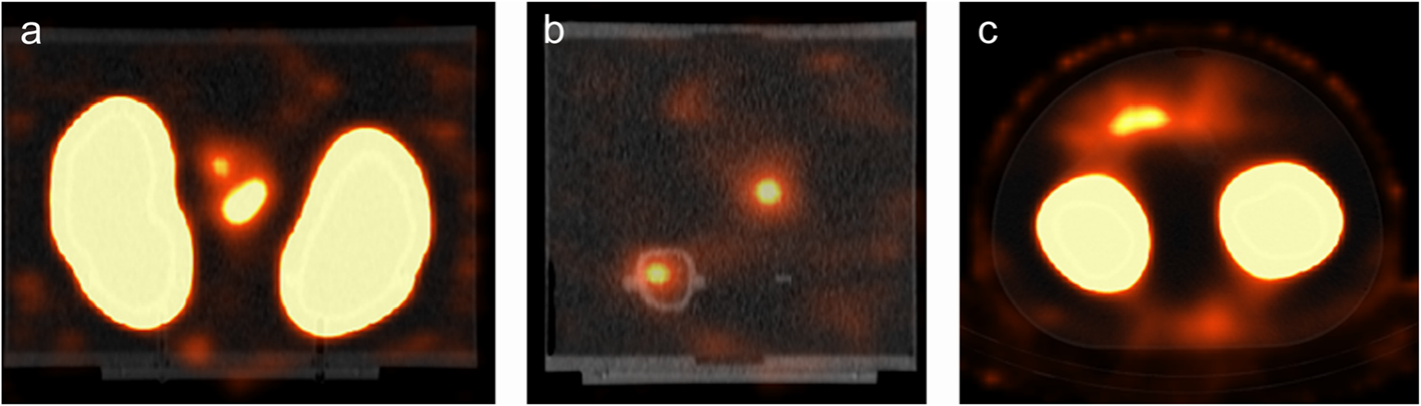
Phantom images from the first experiment reconstructed with Hybrid Recon (**a** coronal view, **b** sagittal view) and Flash 3D (**c** axial view) using 12 iterations and 16 subsets. Scaling was adjusted such the low background intensity is visible. The image reconstructed with Hybrid Recon shows hotspots in the centre between the kidneys (**a**, **b**) with similar intensity as the pancreas between the kidneys, as shown in **b**. In the Flash 3D reconstructed image, a higher intensity region is observed at the back of the phantom. Furthermore, in both images in the background, a low count rim around the kidneys can be observed

In all Flash 3D and Hybrid Recon reconstructions with CC, there seems to be a high intensity rim inside the kidneys, as if there was a cortex in the phantom with a higher activity (Fig. 4a); these images also show a low-intensity shell around the kidneys (Fig. 6). This ring artefact with under- and overshoot appears like the Gibbs phenomenon. In the Flash 3D images, the low-intensity rim is more pronounced than in the Hybrid Recon reconstructions. In the OSEM3D reconstructions without CC and in the MAP reconstruction, this artefact did not appear (Fig. 6b).

Both Hybrid Recon and Flash 3D showed an area in the background in which the intensity is higher than the average background intensity. For Hybrid Recon, this area is located between the kidneys; in Flash 3D, this area is less intense and located more at the back, centrally posterior to the kidneys (Fig. 6). With MAP_smoothing_, this artefact was least pronounced.

### Activity concentration ratios and recovery in the phantom images

**Fig. 7:**
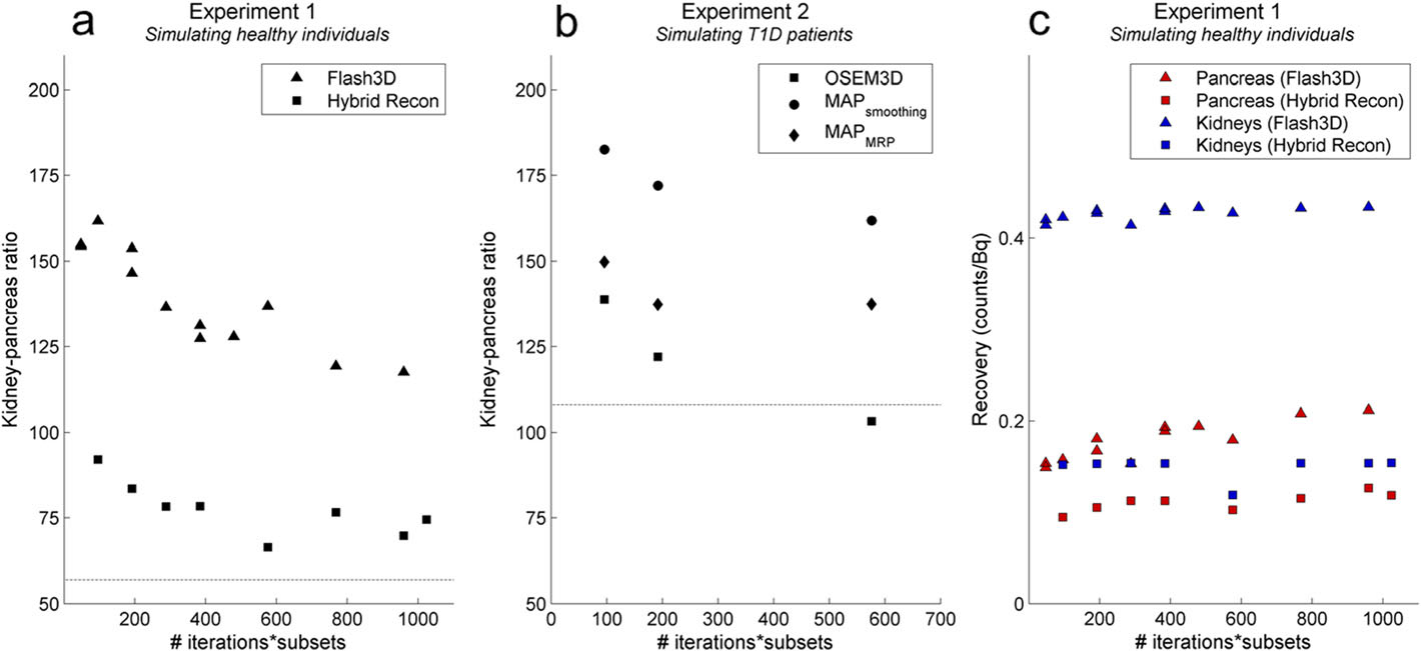
Measured kidney-to-pancreas intensity ratios (as 1/pancreas-to-kidney ratio) in experiment 1 and 2. **a** Measured kidney-to-pancreas intensity ratio for Flash 3D and Hybrid Recon OSEM3D reconstructions in the first experiment. **b** Measured kidney-to-pancreas ratio for Hybrid Recon OSEM3D, MAP-MRP and MAP-smooth reconstructions in the second experiment. Both figures show the measured intensity ratios. The actual activity concentration ratio, based on the real activity concentrations in the 3D-printed organs, is depicted by the *striped lines*. **c** Recovery on the *y*-axis calculated as reconstructed counts/inserted activity (counts/ml)/(Bq/ml). *OSEM* ordered subset expected maximum, *MAP* maximum a posteriori, *MRP* median root prior

Figure 7a, b shows the pancreas-to-kidney intensity ratio for reconstructions with Hybrid Recon and Flash 3D with different numbers of iterations and subsets of the experiments 1 and 2. For both reconstruction programmes, the pancreas-to-kidney ratio increases with an increasing number of iteration times the number of subsets. In experiment 1, the ratio of the Hybrid Recon reconstruction is closer to the real ratio than the Flash 3D ratio.

In experiment 2, the OSEM3D reconstruction leads to more realistic ratios than the MAP reconstructions. For 12 iterations with 16 subsets, the pancreas-to-kidney ratio is 1:122 with OSEM3D, 1:137 for MAP_MRP_ and 1:172 for MAP_smoothing_, while the real ratio was 1:108.

The average pancreas-to-background ratios from respectively the Flash 3D and the Hybrid Recon OSEM3D reconstructions were 1:0.21 and 1:0.037. The actual ratio was 1: 0.005. Within Hybrid Recon, the OSEM3D reconstructions (compared to the MAP reconstructions) lead to ratios that were closest to the actual ratio although large variation can occur due to the low background activity concentration.

Increasing the number of iterations and subsets resulted in increased intensities, demonstrated by the uncorrected recoveries of activities in Fig. 7c. Relatively, this increase was larger in the pancreas than in the kidneys and larger in Flash 3D compared to Hybrid Recon. As an example, comparing 6/16 and 30/32, the relative increase is approximately 35% in the pancreas (Flash 3D 42%, Hybrid Recon 25%) and around 3% in the kidneys (Flash 3D 3.2%, Hybrid Recon 1.4%). In addition, recovery of activity was larger in the kidneys than in the pancreas; this difference was smaller in Hybrid Recon than in Flash 3D (Fig. 7c), corresponding with the more realistic ratios with Hybrid Recon.

### Verification of results on human images

After comparing the results of the different reconstruction protocols, Hybrid Recon with the OSEM3D reconstruction with 6 iterations, 16 subsets, SC, AC and CC was selected for reconstruction of the human data of our clinical trial [1] (Fig. 2b). Small differences can be observed between this Hybrid Recon reconstruction and the initial Flash 3D reconstruction (Fig. 2d). In Hybrid Recon, the intensity within the background and the pancreas is more homogeneous compared in Flash 3D, and a difference in shape of the pancreas is observed. The human SPECT images are similar to the phantom images reconstructed with the optimal Hybrid Recon settings (Fig. 3a) and initial Flash 3D settings (Fig. 3c).

## Discussion

We have developed a phantom that mimics the human situation for ^111^In-exendin imaging, by 3D-printing pancreas and kidney compartments for insertion into the NEMA NU2 image quality phantom casing. The phantom was used for optimization of SPECT reconstruction. Available non-anatomical phantoms mimic the human ^111^In-exendin images of the pancreas and kidneys less accurately. Our 3D-printed phantom led to images that, including artefacts, appeared similar to human ^111^In-exendin images.

The inserted activity in each compartment of the phantom was homogeneously distributed, and therefore, in the reconstructed images, the measured intensity should also be homogeneous. Lower numbers of iterations and subsets gave a more homogeneous intensity, especially in the pancreas. As a result of the incorporated smoothing or median root priors, the images from MAP reconstructions were smoother and more homogeneous within the compartments than the OSEM3D images. Nevertheless, in human, these MAP reconstructions could only be used with a lot of caution because in some human, the true pancreatic spatial distribution of activity need not be homogeneous due to a higher glucagon-like peptide-1 (GLP-1) receptor expression in certain areas of the pancreas, e.g. in the case of an insulinoma. The use of a prior with too much smoothing could reduce the visibility of such regions.

The same applies to filtering. In order to ‘keep the activity in the organ’ and preserve actual hotspots, we used limited filtering (a Gaussian kernel with FWHM of 0.84 cm for Flash 3D and 0.96 cm for Hybrid Recon, corresponding to about twice the voxel size, therewith fulfilling the Nyquist criterion). A larger filter could have been used to obtain smoother images; however, the herewith increased possibility of homogeneous visualization of actually inhomogeneous uptake would have negatively affected the assessment of optimal algorithm performance.

Increasing the number of iterations and subsets improves the recovery of activity in the pancreas (Fig. 7c) and the pancreas-to-kidney ratio (Fig. 7a). It is generally recognized that in iterative reconstructions, more spatial details are visualized when increasing the number of iterations.[14] However, when using too many iterations also, the image noise increases.[15] This leads to increased inhomogeneity in the pancreas, which, again, would not correspond to the actual activity distribution.

A limitation of the phantom is its static nature: In the phantom, the activity is distributed homogeneously within the compartments, and in contrast to patients, there is no movement of organs during the acquisition. In patients, breathing and potentially inhomogeneous distribution of beta cells can induce hotspots within the pancreas.

The Hybrid Recon OSEM3D reconstruction with a relatively low number of iterations and subsets was chosen for the human reconstructions. Extrapolation on Fig. 7a, c indicates that a lower number of iterations and subsets would worsen the pancreas-to-kidney ratio and the recovery of activity in the pancreas.

SC reduces the intensity in the pancreas more strongly than in the kidney. Possibly, an overcorrection in the pancreas area could explain why the actual pancreas-to-kidney ratio could not be reached with the used protocols. Visually, on the SPECT image, the Gibbs artefact, which was introduced by the collimator correction, seems to be more pronounced in Flash 3D (Fig. 6). However, quantitatively, the difference between Flash 3D and Hybrid Recon was less pronounced (see line profile in Fig. 4b); both have a very low number of counts in the surrounding of the kidneys (lower than the background activity).

It appears that the position of the pancreas is more anterior in the Flash 3D reconstructions (Fig. 2c). This might also be caused by the Gibbs artefact that ‘absorbs’ a part of the counts in the pancreas. Therefore, it seems that the pancreas is visualized more anteriorly compared to the images reconstructed with Hybrid Recon.

All corrections, SC, CC and AC, were required to distinguish the pancreas in the second experiment; thus, all corrections are necessary in beta cell imaging with ^111^In-exendin.

So, finally, we selected the reconstruction method that resulted in an optimal visualization of the pancreas and optimal quantitative performance (i.e. pancreas-to-kidney ratio). Hybrid Recon with an OSEM3D reconstruction with 6 iterations, 16 subsets, SC, CC and AC was herewith selected and used for the clinical study. With a different imaging purpose, the optimal settings may be different.

## Conclusions

3D-printing of a phantom that mimics the human pancreas and kidneys was showed to be useful for optimization of ^111^In-exendin imaging. Phantom experiments enabled optimization of the reconstruction protocol in clinical studies. This work contributed to clarify the relationship between SPECT ^111^In-exendin images and the actual situation in humans. So, the use of this custom-made phantom provided essential information for beta cell quantification.
